# Phytochemical Constituents and Derivatives of *Cannabis sativa;* Bridging the Gap in Melanoma Treatment

**DOI:** 10.3390/ijms24010859

**Published:** 2023-01-03

**Authors:** Ellen Schanknecht, Ava Bachari, Nazim Nassar, Terrence Piva, Nitin Mantri

**Affiliations:** 1 The Pangenomics Lab, School of Science, RMIT University, Bundoora, VIC 3083, Australia; 2 School of Health and Biomedical Sciences, RMIT University, Bundoora, VIC 3083, Australia; 3 UWA Institute of Agriculture, The University of Western Australia, Perth, WA 6009, Australia

**Keywords:** *Cannabis sativa*, melanoma, anti-cancer, cannabinoids, terpenoids, flavonoids, inflammation, CBD, THC

## Abstract

Melanoma is deadly, physically impairing, and has ongoing treatment deficiencies. Current treatment regimens include surgery, targeted kinase inhibitors, immunotherapy, and combined approaches. Each of these treatments face pitfalls, with diminutive five-year survival in patients with advanced metastatic invasion of lymph and secondary organ tissues. Polyphenolic compounds, including cannabinoids, terpenoids, and flavonoids; both natural and synthetic, have emerging evidence of nutraceutical, cosmetic and pharmacological potential, including specific anti-cancer, anti-inflammatory, and palliative utility. *Cannabis sativa* is a wellspring of medicinal compounds whose direct and adjunctive application may offer considerable relief for melanoma suffers worldwide. This review aims to address the diverse applications of *C. sativa*’s biocompounds in the scope of melanoma and suggest it as a strong candidate for ongoing pharmacological evaluation.

## 1. Introduction

Cancer, a prolific and devastating disease, is one of the most impacting causes of death and morbidity globally. Regrettably, cancer numbers are increasing as human populations surge and suffer continuous environmental, chemical, and lifestyle-based carcinogenic triggers [1,2,3,4]. This is of specific concern in the case of melanoma. Melanoma is the most deadly and problematic variant of skin cancer. Attributed to ~5% of all skin cancers, melanoma is estimated to cause more than 75% of skin cancer-related deaths [5,6]. It is characterised as a neoplastic disorder affecting melanocyte cells in the epidermal layer of the skin [7]. These pigment cells aid in the protection of lower epidermal layers through the production of melanin [7]. Melanomas of all types have heightened metastatic potential and may generate tumours throughout the body via distant, satellite, or local invasiveness [8]. Pharmacological treatment options include both mono- and dual-therapies of small-cell kinase and immune checkpoint inhibitors [9,10]. The genesis of these drugs has seen major improvements in melanoma treatment in the past ten years. However, the metastatic and insidious nature of this disease makes long-term resistive treatment difficult. Due to the heterogenicity and plasticity of melanoma tumorigenesis, the use of combined therapies is needed [11,12], and is becoming standard care for metastatic patients [13]. However, heavily impairing side effects and unacceptable toxicities in patient populations all weigh unfavourably on the efficacy of current treatments [14,15,16,17,18]. There is a clear need for a multitargeted approach that is not hindered by the contraindications of present single-drug options. It is speculated that this approach is to be found in plant-based treatment. 

*C. sativa* (*Cannabis sativa* L., *Cannabaceae*) has medicinal roots dating back at least 1800 years to early Chinese medicinal texts that describe the use of its seeds as a laxative [19,20]. Other less substantiated accounts present the use of this plant dating back much further in many cultures around the world, 5000 years in some estimations [21]. Research into the true scope of the medicinal profile of cannabis and its biochemical constituents is still ongoing. *C. sativa* produces over 500 bioactive compounds [22], of which an increasing number hold potential for the treatment of disease; and as frameworks for the development of novel drugs [22,23,24]. Chief among these are cannabinoids: Δ^9^-tetrahydrocannabinol (THC) and cannabidiol (CBD). THC—the psychoactive agent of cannabis. THC and its synthetic analogs are used to reduce emesis, stimulate appetite in the treatment of HIV-induced anorexia, and for the management of spasticity disorders [18,25,26,27,28,29]. Furthermore, CBD has diverse medical value due to its anti-inflammatory, neuroprotective, and palliative utility [30]. Isolation of these key cannabinoids and the administration of their synthetic counterparts has generated several approved medicinal products that have supportive impacts on cancer patient care. Dronabinol (Morabinol^®^), Sativex^®^, and Nabilone^®^ along with whole cannabis compounds including dried flowers and their extracts, make up most prescribed cannabis pharmaceuticals, “cannaceuticals”. A study of 10,000 German patients prescribed cannabis products, demonstrated that Dronabinol was most frequently administered in 65% of study participants, followed by cannabis flowers at 18% [31]. The primary uses of these cannabis products were to manage pain in 73% of participants, spasticity in 10%, and anorexia in 6% [31]. However, the application of *C. sativa* in the direct—rather than adjunctive—treatment of cancer has limited clinical examination. Whole cannabis administration, though the most common route by which cannabis is consumed for its global nociceptive and psychotropic exploits has had insufficient exploration to determine its long-term or chronic use impact. Investigation of the diverse potential of combined and individual components of cannabis warrants critical discussion. In the case of melanoma, the potential of cannabis to allow for a multitargeted treatment option is a complex topic that will be discussed herein. 

## 2. Factors Effecting Melanoma Classification, Onset, Progression, and Risk

Melanoma progression commonly consists of; first, local spreading, followed by invasiveness to surrounding tissues and finally metastasis to unrelated tissues within the body [12]. This section will address the key factors which denote the clinical staging and characterisation of melanoma and how the histological, genetic, extra- and intra- environmental factors of this cancer denote its risk of onset and progression. 

### 2.1. Subtypes of Melanoma 

Melanoma has four location-based subtypes: (1) cutaneous, (2) acral (occurring in the nailbeds, palms and foot soles), (3) mucous membrane derived, and (4) uveal (of the eye and surrounding ocular tissues) [5,7,9]. The most prevalent, CM, is known to arise within melanocytic nevi (moles) which undergo genetic changes that induce pro-cancerous pathways. However, mutational changes are not isolated to nevi and may occur de novo from any melanocytic cell. Further classification of cutaneous variants of melanoma are determined by clinical presentation of the primary lesion [32]. Histological classification of tumour types include: acral lentiginous, lentigo maligna, nodular and superficial spreading. Rarer non-cutaneous variants are often deadly due to their less detectable and difficult to treat nature [33]. Many factors thus affect the characterisation of this cancer, some imparting higher risks.

### 2.2. Risk Factors of Melanoma 

The number of melanoma cases across 185 countries has risen by ~69% since 1992 [34,35]. Factors including geographical location, gender, age and familial history of the disease all influence the potential for melanoma occurrence [36,37,38]. There are significantly lower survival indices in those aged between 50–59, those who are over 80, have lower-income socioeconomic backgrounds, are Black patients and display more advanced staging and histological presentations of nodular and acral lentiginous melanoma; such as, ulceration and higher serum lactate dehydrogenase (LDH) levels [39]. 

Ultraviolet (UV) light, especially UVB exposure has prevalence as a contributing environmental carcinogen [40,41,42]. Chronic sun exposure particularly in early childhood, in fair skin and hair type individuals, poses the highest evident risk of melanoma onset [43,44]. Exposure to UVB resulting in sunburn, particularly intermittent and extensive sunburn resulting in blistering is correlated with an almost doubled risk of melanoma diagnosis [45]. Moreover, UVA-type light penetrates deeper into the dermis and is mainly attributed to peripheral carcinogenic effects through the creation of reactive oxygen species (ROS) causing significant DNA damage and ongoing inflammation. 

#### 2.2.1. Reactive Oxygen Species 

Reactive oxygen species (ROS) such as hydroxyl free radicals (OH^−^), anion superoxides (O_2_^−^), peroxyl (RO_2_^•^), hydroperoxyl (HO_2_^•^) normally exist as metabolic by-products of regular cellular function [46,47]. However, the under-management of their production by cellular antioxidant mechanisms may lead to the induction of apoptosis pathways and the dysregulation of gene expression. Apoptosis may arise from endoplasmic reticulum (ER) stress induced accumulation of ceramides and proinflammatory factors.

Oxygen free radicals such as those mentioned above are highly reactive due to unpaired electrons present in their outermost shell [46,47]. ROS play a significant role in cell homeostasis due to their role in electron exchange, which is facilitated by their chemical reactivity. However, ROS over-expression is known to effect cellular metabolism via the induction of oxidative stress, a state often resulting in cellular damage and death.

#### 2.2.2. Chronic Inflammation

**Figure 1:**
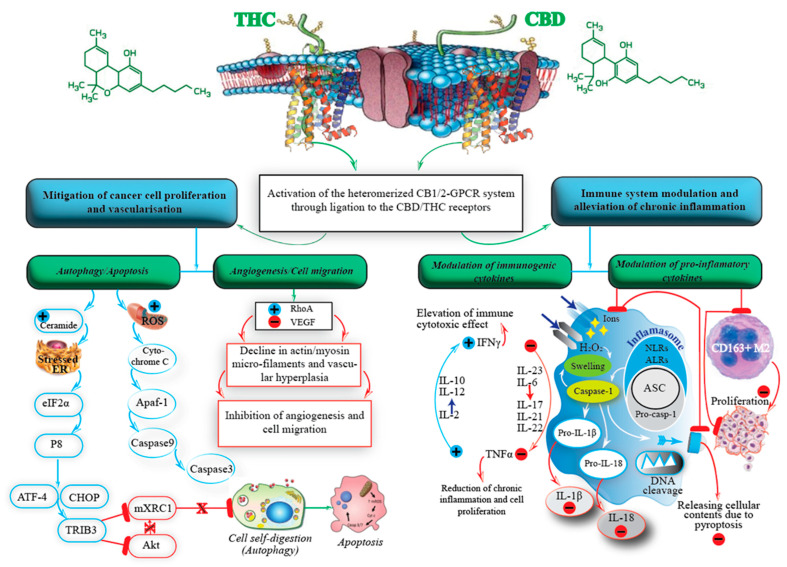
Metabolic pathways of melanoma and their response to cannabinoids exposure. Red indicates inhibition and blue indicates induction of these pathways.

Chronic inflammation in the skin is related to several chronic medical conditions such as: ectopic dermatitis, psoriasis and severe eczema. These inflammatory disorders can lead to elevated levels of ROS in epidermal tissues. In addition to the ROS-related factors, chronic inflammation can increase the incidence of melanoma by 20%. This effect is associated with an imbalance of immunological cytokines generated by higher levels of secretions from inflammasomes, IL-1ꞵ, IL-18, and IL-6 resulting in elevation of tumour necrosis factor alpha (TNFα) and a reduction in interferon gamma (IFNγ) (Figure 1). Thus, mitigating oxidative stress and subsequent inflammation poses a desirable target to lower risk conditions correlated with the induction, progression and self-feeding cancer environment of melanoma. 

#### 2.2.3. Reducing Oxidative Stress Risk

Products with antioxidant capacities are desirable for their diverse ability to disrupt ROS imbalances within the cell. Antioxidants act through a variety of mechanisms including; (1) physical blockage of ROS from critical cellular sites; (2) sequestering of orphan electrons to “diffuse” ROS; (3) catalytic functionality that may transport or offset ROS through binding interactions; (4) inactivation of metal ions which stalls ROS production; and (5) breaking of the ROS production chain through OH group interactions [48]. Cannabis has a diverse content of phenolic compounds many of which have significant ROS scavenging and disrupting capacities, primarily as chain-breakers due to the prevalence of their hydroxyl group expression. Prevention of oxidative stress by utilising antioxidants has critical implications in mitigating risk from environmental factors including UVR via the aforementioned mechanisms. 

### 2.3. Genetic Influences of Melanoma 

Differences in genetic origin of melanoma have significant impacts of responsiveness of the disease to treatment. Certain genetic and histological variants are more associated with low recurrence-free survival due to metastatic progression [49,50]. The risk associated with these variants correlates to extra- and intra-environmental factors, inducing pro-cancerous genetic expression [51]. 

Moreover, such heterogenicity poses diagnostic and treatment challenges. These include: (1) lack of universal genetic markers; (2) genetic multivariance within individual tumours; (3) high opportunity for treatment resistance; and (4) unpredictable responsiveness of different genotypes to cancer therapies [52,53,54,55]. 

There are several common genetic loci associated with the development of cutaneous and non-cutaneous melanomas. These primarily centre around the activation of *RAS-RAF-MEK-ERK* and *P13K-Akt* signalling pathways. Mutations of *V600E* and *K* result in *v-Raf murine sarcoma viral oncogene homolog B1* (*BRAF*) and mitogen-activated protein kinase kinase (*MEK*) mutational variants. The most common mutation *V600E*, a substitution mutation of valine by glutamic acid at position 600, is attributed to ~50% of all diagnosed cases of CM [51,56,57]. 

Targeted therapy is the primary pharmacological treatment strategy for these genetic melanoma types [10]. The constitutive expression of these genes leads to an upregulation of the *mitogen-activated protein kinase* (*MAPK*) pathway in these melanomas, resulting in an excess of proliferative, invasive and immuno-avoidant cancer characteristics [58,59]. Other genomic subtypes include *neuroblastoma rat sarcoma viral oncogene homolog* (*NRAS*), and *neurofibromatosis type 1* (*NF1*) mutations. *NRAS* and *NF1* have yet to result in the development of gene specific drugs [60,61]. Familial cutaneous malignant melanoma, comprising ~10% of all melanomas, displays mutations of the tumour suppressor gene *Cyclin Dependent Kinase Inhibitor 2A* (*CDKN2A*) [62,63]. The latter has a role in the production of several important tumour suppressor proteins. For example, p16 which stops *Cyclin Dependent Kinase 4* (*CDK4*) and *6* (*CDK6*) from stimulating cell proliferation, and p14 which is involved in the prevention of the apoptosis and senescence regulating protein p53’s destruction. *CDKN2A* and either *BRAF* or *NRAS* (non-simultaneously) mutations may occur concurrently within one melanoma tumour, contributing to difficulty in ongoing and initial tumour treatment. Moreover, administration of targeted therapies that are not suited to the genomic expression of the total body-scale tumour environment, including those which may develop as distant ITMs, may induce worse outcomes [64]. This makes targeted therapy a hit-or-miss treatment course, dependent on, not only accurate genotyping of several locations of the primary tumour, but subsequently the ongoing assessment of secondary metastases. 

Furthermore, many additional genetic mutations including *Telomerase reverse transcriptase* (*TERT*), *Microphthalmia-associated transcription factor* (*MITF*) and other auxiliary genetic contributors complexify the dynamics of transcriptional factor expression resulting in downregulation of tumour suppressor genes factors [65]. This includes the crucial role of microRNAs (miRNAs) which regulate drug resistance of melanoma and influence its angiogenesis, progression and severity [9,53,66].

## 3. Treatment Outlooks for Melanoma 

Primary treatment of melanoma is mostly conducted through surgical intervention which has curative success if directed early enough in the cancer’s lifecycle [67]. Unfortunately, early detection is not always possible, and several subtypes of melanoma are more highly associated with rapid metastasis to surrounding tissues [68]. Moreover, surgery may produce debilitating disfigurement, pain and high risk of infection. 

### 3.1. Surgical Implications 

Melanoma risk is not limited to genetic predisposition and UV exposure. Indeed, the risk of malignancy may be induced by a multitude of traumatic skin events. Frequent and significant surgical resection events that result in repeated scaring, pose long-term risk for subsequent malignant transformation of damaged skin. For example, increased incidences of malignant transformation of squamous cell carcinoma (SCC) occurs in 12% of patients with extremity chronic osteomyelitis (COM) [69]. The induction of post-surgical metastases are correlated with surgery related stress [70]. As well as, post-surgery immune suppression through reduced Natural Killer (NK) cell cytotoxicity, which impairs the clearing of tumour related emboli [71]. 

Furthermore, associated post-surgical inflammation is an ongoing risk. Inflammation is one of the ten major indicators for cancer [72]. Relatedly, persistent inflammation in many diseases is correlated with worse prognosis and higher instance of disease onset. Epidemiological data suggests that up to 20% of all cancers begin with direct relation to states of chronic inflammation [73]. The complex mechanisms of inflammatory dysregulation in the skin and consequent risk of malignant occurrences may be able to be better understood (or perhaps mitigated) through the exploration of endocannabinoid receptor signalling in the skin environment [74]. Furthermore, the targeted action of cannabis phytocompounds to these sites could reduce the impact of this dysregulation by reducing inflammation and by influencing the recruitment of immune cells such as NK [75]. 

### 3.2. Pharmacological Treatments of Melanoma

Pharmacological outputs for melanoma treatment have primarily comprised of targeted and immune therapies. Developments of these treatments was necessitated by poor increases to survival rate for comprehensive medication management (CMM) with traditional chemotherapeutic strategies [76,77]. Developed in 2012, targeted *BRAF* kinase inhibitor Dabrafenib demonstrated significant PFS improvement of 5.1 months compared to 2.7 compared to chemotherapy drug dacarbazine [78]. The development of other targeted therapeutics and their use in combination, was a gamechanger in improving outcomes for CMM patients [79,80,81,82,83,84,85]. For patients without the *BRAF* genotype, immunotherapies have shown promise as an alternative treatment [84,85]. Despite these advancements, current treatments induce states of inflammatory stress and have impairing side-effects. The pros and cons of current pharmacological treatments will be discussed in the subsections below. 

#### 3.2.1. Targeted Therapy

Discovery of *BRAF* mutant genes and their impact on progression and metabolism in melanoma has led to the development of small-molecule inhibitors. *BRAF* inhibitors (BRAFi) and *MEK* inhibitors (MEKi) regulate overexpression of related kinases, effectively halting growth and continuance of melanoma via disruption *MAPK* pathway signalling in cancerous cells [86,87]. Additional treatment options have subsequently been developed for the treatment of non-*BRAF* and *BRAF* mutant individuals alike, with varying success (Table 1). 

#### 3.2.2. Immune Therapy 

Currently, two categories of immune-based therapies are employed in the treatment of melanoma. These include programmed cell death protein 1 (PD-1) and cytotoxic T lymphocyte associated protein 4 (CTLA-4) inhibitors [88]. These antibodies inhibit certain receptor targets that disguise cancer cells from the body’s immune system. Essentially, these inhibitors de-camouflage cancerous tissues and thus allow for their destruction via immune cells [88]. The emergence of these targeted immune therapies has had a significant improvement for patient outcomes compared to early treatments [89]. Furthermore, these antibodies have comparatively superior toxicity profiles from a chemotherapeutic perspective. However, toxicity from their administration may still heavily impact a patient’s wellbeing, with multiple patients unable to sustain treatment due to personal toxicity and adverse events [90]. Existing pharmacological options have gaps in treatment effectiveness especially for advanced forms of this disease. Variations that display mutable resistance to common drug treatment options are clear [52,91]. For these reasons, alternative medication and treatment options are sought. This is especially relevant in cases of [92,93] *BRAF wild-type* (*BRAF^WT^*) that is, non-*BRAF* mutated individuals and immune treatment resistant melanoma strains [92,93,94,95]. Modern drug options face downfalls in their long-term application from the perspective of treatment resistance, overall success rate, significant side-effects and ongoing financial burden to patients [96]. To combat these limitations multi-drug administration has been employed; with the goal of alleviating said drug resistance and promoting longer survival [97].

#### 3.2.3. Multi-Drug Administration Effectiveness 

In the case of *BRAF* mutant melanoma, BRAFi and MEKi are used in combination to elicit higher treatment potency (Table 1). This is conducted via the administration of inhibitors that target different stages of the same pathway to prevent its reactivation [98]. The efficacy of dual versus mono-treatments has been well established in COMBI-v and COMBI-d trials [91,99], with a 22% progression free survival (PFS) in combined Dabrafenib/Trametinib (BRAFi/MEKi) versus 12% in mono Dabrafenib administration [83]. Three-year OS was also significantly improved at 44% compared to 32% in the same trial [100]. In combined treatments of BRAFi Vemurafenib and Dabrafenib, 12-month OS was significantly improved compared to mono-treatments at 72% and 65%, respectively. Patients where significantly more likely to achieve durable three-year survival with the administration of these dual therapies [84]. 

Combined targeted and immune therapies are emerging with variable efficacy (Table 1). Combined Ipilimumab and Nivolumab saw a drastic improvement in objective response compared to that of Ipilimumab alone, at 61% response rate compared to 11%, respectively [101,102]. However, adverse response was more highly associated with combined therapy, with 54% of patients experiencing higher than grade 3 (severe) side-effects compared to 24% from Ipilimumab alone [101,102]. Concurrently, four-year follow-up outcomes for stage III trials of combined versus individual treatments of Nivolumab and Ipilimumab showed PFS of 11.5, 6.9 and 2.9 months for combined, Nivolumab then Ipilimumab, respectively [84]. However, grade 3–4 side effects were notable in combined administration with 59% experiencing adverse impacts compared to 22% and 28% for individual Nivolumab and Ipilimumab [84]. 

Triple drug therapies for combined immune checkpoint inhibitors and BRAFi/MEKi have also been tested [82,103,104,105,106]. However, triple combinations are associated with higher toxicity profiles [82,90,106,107,108,109], with several being associated with unacceptable toxicities and early trial termination [82,106]. Specifically, inflammatory adverse events such as colitis, hepatitis and pyrexia are common; severe gastric distress including diarrhoea, vomiting and nausea generate intolerable treatment exposure conditions for some patients [82,90,106,107,108,109]. 

**Table 1:** Combined chemotherapeutic therapies, their effect on progression free survival (PFS) and overall survival (OS) of patients and associated side effects of their administration in clinical trials for melanoma.

Treatment	Patient Population	Effect on Survival	Side Effects	Reference
Dabrafenib + Trametinib	Unresectable or metastatic melanoma with BRAF^V600E/K^	↑ Treatment response rate ↑ PFS in patients with advanced melanoma	Pyrexia, nausea, arthralgia, fatigue, diarrhea, chills, vomiting, headache	[83]
Nivolumab + Ipilimumab	Previously untreated advanced melanoma	↑ OS ↑ PFS	High occurrence of gastrointestinal (diarrhea, colitis), skin-related (pruritus, rash) and pyrexia events	[84]
Vemurafenib + Cobimetinib	Previously untreated, unresectable locally advanced or metastatic BRAF^V600^	↑ PFS with BRAF^V600^ metastatic melanoma. ↓ Incidence of cutaneous secondary cancers	Rash, diarrhoea, photosensitivity, hepatic-enzyme abnormalities	[82]
Encorafenib + Binimetinib	Unresectable or metastatic melanoma with BRAF mutations	↑ OS ↑ PFS	Nausea, diarrhoea, fatigue, arthralgia, omitting, pyrexia, and increased aspartate aminotransferase (AST)	[81]
Nivolumab + Relatimab	Previously untreated advanced melanoma	↑ PFS	Well tolerated with a manageable safety profile	[110]

### 3.3. Limitations of Existing Treatment Options 

Although great strides in the development of melanoma drugs have occurred in the last 10 years, there are several pitfalls associated with their administration. These are (1) treatment resistance; (2) intolerance and toxicity; (3) low long-term survival outlooks; and (4) financial cost. 

#### 3.3.1. Treatment Resistance 

Resistance in melanoma is attributed to several different mechanisms: (1) reactivation of signalling pathways; (2) modification of drug target genes; (3) circumvention of signalling pathway alteration via alternative pathway activation; (4) distant cancer emergence of genetic dissimilarity to the primary tumour and (5) the potential for cancer dormancy [97]. As indicated above, melanoma may express multiple genotypic variants even within the same tumour. The capacity for the drug resistant characteristics of melanoma may be derived from genetic mutations in any of its cancer contributing loci, in addition to the manipulation of oncogenic miRNA regulation. This multiplicity of defence sets an immense long-term challenge regarding the resistant behaviour of melanoma. Notably, this has resulted in severely limited long-term outlooks in metastatic individuals. Evidenced by, the five-year survival rate in patients with stage IV metastatic melanoma only reaching 23% [101]. 

#### 3.3.2. Intolerance and Toxicity 

Associated side-effects in application of current melanoma treatments include cutaneous disorders, fever, fatigue, nausea, inflammatory disease and many others. This limits the efficacy of long-term administration of these drug combinations leading to forced cessation of treatment or intermittent treatment regimens [16,17,18]. 

In terms of drug effectiveness, BRAFi and MEKi such as Dabrafenib and Trametinib have only a 50% estimated response rate. Side-effects are often severe, with most clinical trials indicating Grade 3 and 4 adverse effects in over 50% of the patient population with combined drug administration of targeted and immune therapies [90,107,108]. Immune therapies such as PD-1 and CTLA-4 inhibitors Nivolumab (anti-PD1) and Ipilimumab (anti-CTLA-4) have a 60% response rate in patients [103]. However, CTLA-4 administration has a high instance of inflammation resulting in colitis, hepatitis and many other inflammatory symptoms [111,112]. The complex surgical and pharmacological morbidities in the treatment of melanoma all contribute to its low long-term treatment responses. 

#### 3.3.3. Low Long-Term Survival Outlooks 

Due to these factors, current treatment regimens result in poor five-year survival outcomes for malignant diagnosis. Most metastatic melanoma sufferers do not survive beyond three years of treatment [113,114]. Additionally, there is a 13.4% rate of melanoma reoccurrence within two years in high-risk primary tumours [4,5]. 

#### 3.3.4. Treatment Cost

Further treatment limitations notably include their high cost [96]. Close to 80% of the cost of melanoma treatment was spent on drug treatments alone [14]. Furthermore, the cost of treatment in Australia rises tenfold in individuals with Stage III/IV diagnoses [14].

### 3.4. Bridging the Gap in Melanoma Treatment

The treatment-evasive nature of melanoma, in conjunction with the total scope of its clinical pitfalls, clearly necessitate the application of new pharmacological and adjunctive treatment options. The genetic and microenvironmental complexities of melanomas have contributed to the ineffectiveness of single drug treatments. Moreover, emerging multidrug resistant tumours exist in patient populations, ultimately resulting in no further clinical options for these patients and near certain death [101]. Palliative support then becomes the only option for these sufferers. Holistic multi-targeting pharmacological products are needed to tackle the immense challenges that melanoma presents. Biocompounds from *C. sativa* may answer many of the diverse requirements for ongoing melanoma treatment, not only through its adjunctive capacities for pain, nausea and inflammation management, but also through direct anti-cancer actions related to the endocannabinoid system, discussed below. 

## 4. The Endocannabinoid System

Within human and other mammalian systems there is a network of receptors that respond to endogenous cannabinoids (endocannabinoids) and comprise what is known as the endocannabinoid system [115]. This system has a diverse role in many aspects of mammalian functioning. It is known to exert regulatory functions on the cardiovascular, immune and reproductive systems [116,117,118], as well as having various effects on metabolic processing. Primarily, the endocannabinoid system holds a crucial neuroregulatory and homeostatic role in the human body. 

The first hallmark discovery for the endocannabinoid system was triggered by an interest in THC and a slew of its synthocannabinoid mimics. The first cannabinoid receptor (CB1) was defined through pharmacological examinations of the binding behaviours of these compounds in 1988 [119].

Evolving knowledge of the endocannabinoid system shows that it consists of a network of receptor targets and the enzymes responsible for their degradation and transportation. Endocannabinoid receptors are G-protein coupled receptors that are members of the G-protein superfamily. They currently consist of cannabinoid receptors CB1 and CB2. These canonical receptors are leading targets for drug administration of *C. sativa* and its products. CB1 and CB2 share a high proportion of homology, with 44% genetic sequence similarity [120], yet each represent different roles and primary areas of involvement in the body. CB1 is more commonly expressed in neural tissues and is found in higher quantities in the central nervous system. CB2, while also present in the brain, is more notably found in immune cells. Fundamentally, both CB receptors could be said to be constitutively expressed throughout all body systems, due to the omnipresent nature of immune cell involvement [121]. The ubiquitous characteristics of these receptors indicate them as a complex target for disease regulation. However, little is known regarding their complex metabolic and epigenetic influence. 

In addition to wider regulatory functions, endocannabinoid receptors have been found to be constitutively expressed in many cancerous tissues of interest, including melanoma [122]. Furthermore, regulation of endocannabinoid system tone—that is, the levels of endogenous cannabinoids, their enzymes and transport proteins—may have a critical role in chronic inflammatory diseases through the regulation of inflammatory cytokines and the modulation of ROS (Figure 1) [123]. The intricate role of the endocannabinoid system presents an intriguing pharmacological target. However, a full picture of the role of the endocannabinoid system in human functioning is still limited. 

## 5. Cannabis and Its Constituents 

In the context of its societal application, the utility of *C. sativa* may be broken into several facets, namely, [9] its psychoactive potential which leads to its recreational usage; its practical and industrial potential as a fibre, edible seed and ingredient of biofuel [5]; and its multimodal potential as a medicinal compound. The direct and adjunctive application of *C. sativa*, as a whole product and from its phenolic constituents for the treatment of melanoma will subsequently be addressed. 

### 5.1. Classification of Cannabis 

*C. sativa* is a highly versatile plant that displays significant levels of phenotypic variability. In the street vernacular, cannabis or marijuana (as it is more commonly known) is distinguished into two separate subspecies, *C. sativa* and *Cannabis indica* (*C. indica*) [124]. Genetic evidence suggests that the chemical variability and phenotypic uniqueness of observed cannabis populations do not define a special separation. Instead, genetic profiling indicates that cannabis is an extremely structurally diverse plant that demonstrates high levels of genetic polymorphism. Indeed, it is a well-known feature of the cannabis plant to be highly cross bred with other so-called “species” of the plant. Therefore, the phenotypic variations of these plants are not considered to delineate them as separate species or subspecies by traditional classification [124]. However, this point has been routinely contested in the literature and discussions are ongoing. Many other flowering plants may also breed sexually to produce viable offspring even those with hundreds of phenotypically distinct varieties, such as the African violet. It becomes problematic therefore to denote what are visually, chemically and functionally unique varieties of such highly genetically plastic plants. Visual and geographical differences in the case of cannabis tentatively define four “species”: *C. sativa*, *C. indica*, *Cannabis ruderalis* (*C*. *ruderalis*) and *Cannabis afghanica* (*C. afghanica*) [125]. Strong taxonomic arguments against the inclusion of the latter two; including, lack of significant genetic difference and the production of viable interbred offspring have been addressed in previous literature [126]. For the purpose this review, chemical variability “chemovar” rather than taxonomic definition has greater importance in the scope of melanoma treatment and thus will be discussed herein. 

Current classification of cannabis into defined groups is limited to three generalised labels, including: hemp, with less than 1% detectable THC content; high THC variants, and high CBD variants. Synthetic analogues of cannabinoids are also an emerging group for consideration. Differences in psychoactive effects of *C. sativa* are likely due to the broad opportunity for variation in the production of its complex array of chemical compounds. 

### 5.2. Classification of Cannabinoids 

Cannabinoids are defined by several means: structurally, as heterocyclic ring-containing formations; relational similarity to primary cannabinoid structures; and from their direct and indirect actions against the endocannabinoid system. Cannabinoid types discussed in this review are listed in Table 2, detailing their individual and/or synergistic effects tested in vivo and/or in vitro. The following cannabinoid types are discussed herein: (1) endogenous cannabinoids (endocannabinoids); (2) natural phytocannabinoids; and (3) synthetic analogues of cannabinoids herein named “synthocannabinoids”. 

### 5.3. Endogenous Cannabinoids 

The endocannabinoids that have had the most detailed examination are derivatives of arachidonic acids: N-arachidonoylethanolamine (AEA) and 2-arachidonoglycerol (2-AG) [115]. Additional influencers of endocannabinoid metabolic action include the N-acyleethanolamines (NEAs): palmitoyethanolamide (PEA) and oleoylethanolamide (OEA) [127]. In combination, these compounds play a vital role in a variety of physiological and systemic responses. Endocannabinoids and NEAs circulate in human blood and are also found in many human fluids and tissues such as hair, saliva, breast milk, saliva, semen and amniotic fluid [128]. The concentration of endocannabinoids in different areas of the body, both from a systemic perspective in the blood and plasma and locally within different tissues of the body, is dynamically regulated in response to intracellular and external stimuli. Influencers of this system are known as “cannabigerics”. 

### 5.4. Phytocannabinoids 

Cannabis produces a highly diverse array of isoprenylated resorcinyl polyketides that are called phytocannabinoids. However, the complete definition of phytocannabinoid in the sphere of cannaceutical understanding is a difficult one. Cannabinoids are highly structurally homogenous; however, they differ significantly with respect to the orientation and chemical differences of each of the three moieties which define their most common structures. These moieties consist of a resorcinyl core flanked by an isopentyl residue and sidechains of different variation. Traditionally, phytocannabinoids have been discussed primarily in relation to the most infamous of cannabinoid structures, Δ^9^-THC, as well as the canonical CB receptors. 

Structural consensus is inconsistent throughout the literature, with over five numbering systems for the different cannabinoid types [125]. This holds significant difficulty for the ongoing structural definition of these compounds. The traditional three-letter naming vernacular common to major neutral cannabinoids has existed since the 1960s. This system remains largely unchanged even through the discovery of over 200 additional phytocannabinoid compounds. Moreover, the limitation of current naming definitions neglects those cannabinoids not isolated or found from cannabis. Indeed, there are ongoing discoveries which indicate unique concentrations and alternative cannabinoid compounds in other plants and perhaps more interestingly, in fungi and bacterial species [129,130].

There are arguments to define phytocannabinoids based on their structural similarities and dissimilarities compared to the main classes of cannabinoids, namely: THC, CBD cannabigerol (CBG), cannabinol (CBN), cannabichromeme (CBC), cannabidivarin (CBV) and other minor cannabinoids such as cannabicyclol (CBL) and cannabielsoin (CBE) [131]. This is based on the popularity and commonality of these structures and their homology for most of the forms of phytocannabinoids currently elucidated. Moreover, numbering and IUPAC naming of these compounds have not been consistent, making definition by this basis currently too unspecific. 

Additional variations of these so called “natural” incidences of the cannabinoid compounds include their acidic or carboxylated variations as well as a variety of their isoforms relevant to this discussion [25,131]. Heat and other high energy stress results in the decarboxylation of the acidic formations of these cannabinoids to more commonly medicinally acknowledged forms. Promisingly, recent studies have implicated cannabidiolic acid (CBDA) for its anti-cancer effects in colorectal cancer via the induction of apoptosis and cell cycle arrest [132]. LNCaP cells have also been shown to have low cell survival when exposed to high concentration carboxylated natural phytocannabinoid extracts such as Δ^9^-THCA, CBDA and others [133]. Emergent canna applications of minor cannabinoids and cannabinolic acids require further study. Preliminary explorations show promise for future applications of these products, especially in the case of cannabigeriol (CBG) addressed in the next section.

### 5.5. Synthocannabinoids

Administration of synthocannabinoids is a commonplace activity in research since the discovery of THC in 1964 by Raphael Mechoulam [134]. The discovery of key cannabinoids THC and CBD initiated extensive in vitro research performed for the potential of these compounds and their individual administration. Emergent single synthetic drug agents have since been approved for a multitude of pathologies. Primarily, agents such as Nabilone^®^ are approved for their antiemetic, anti-anorexic and nociceptive properties [135,136,137]. The wider palliative utility of such products has demonstrated encouraging adjunctive capacity in cancer sufferers. The growing use of synthetic products is becoming widespread as public perceptions shift. For example, approval of combined THC:CBD 1:1 spray Sativex has recently been approved for use in Australia [138]. A benefit of synthetic derivatives is the specificity and purity of their compositions. This is a factor that has largely been sought in modern pharmacological outlooks for several reasons, namely: (1) the reproducibility of consistent treatment concentrations; (2) purity of such products is easily determined using analytical measurement; (3) large quantities may be produced via chemico-industrial practices and (4) drug intractability is theoretically lessened by the administration of single or few drug products [139]. Unfortunately, many positive aspects of this type of drug design and administration limit their capacity for holistic treatment of highly complex diseases such as cancer. Combined synthocannabinoid administration and whole cannabis formations, rather than single cannabinoids, have demonstrated higher efficacy for the treatment of cancer [16,140].

**Table 2:** Individual and combined treatments of broad cannabinoid-type compounds. Tested range of treatment concentrations, associated effect and cannabinoid receptor involvement for in vitro and In vivo studies of melanoma cell types are displayed herein.

Cannabinoid	Cell Type	Mutation	In Vitro/ In Vivo	Dose	Effect	Receptor	Reference
**Exogenous**							
**THC**							
**Synthetic**	A375, SK-MEL-28, CHL-1	BRAF, CDKN2A and TERT (A375) and BRAF, CDK4, CCLE, EGFR, PTEN, TERT, TP53 (SK-MEL-28)	In vitro	1–5 μM	Primary melanocytes unaffected up to 6 μM THC ↓ Cell viability ↑ Apoptosis ↑ Beclin1 and Ambra1-independent autophagyRegardless of BRAF mutational status	N/A	[141]
	CHL-1 injected athymic mice	CDKN2A, MAPK3, TERT, TP53	In vivo	15 mg kg^−1^	↓ Cell viability ↓ Tumour growth ↑ Apoptosis ↑ Antitumor response	N/A	[141]
	HCmel12 and B16	DMBA-induced HGF-CDK4^R24C^ melanoma (HCmel12) and spontaneous mouse mutant (B16)	In vitro	5 and 10 μM	No effect	CB1, CB2 Low expression	[142]
	HCmel12 injected HGF-CDK4^R24C^ and Cnr1/2^−/−^ mice and B16 injected HGF-CDK4^R24C^ mice	DMBA-induced HGF-CDK4^R24C^ Cnr1/2^−/−^	In vivo	5 mg/kg body weight	↓ Tumour growth ↓ Inflammatory response ↓ Infiltration of CD45+ immune cells Tumour angiogenesis unaffected B16 and HCmel12 Cnr1/2^−/−^ mice not significantly affected	CB receptor-dependent affect for HCmel12 injected mice, CB1, CB2	[142]
**Plant based**	B16, A375, MelJuso	Spontaneous mouse mutant (B16), BRAF, CDKN2A and TERT (A375), HRAS and NRAS (MelJuso)	In vitro	1, 2, 2.5 and 3 μM	↓ Viability ↓ Proliferation ↑ Apoptosis No effect on mouse melan-c and human Hermes 2b healthy melanocytes	CB1, CB2, Comparable CB1 expression in mouse melan-c and human Hermes 2b healthy melanocytes	[143]
**CBD**	B16	Spontaneous mouse mutant	In vitro	0.0016–0.2 mg/mL	↓ Cell growth	N/A	[144]
	B16F1, A375	Spontaneous mouse mutant (B16), BRAF, CDKN2A and TERT (A375), HRAS and NRAS (MelJuso)	In vivo	5 mg/kg twice per week	↑ Survival duration ↑ Quality of life ↑ Movement ↓ Tumour growth	N/A	[121]
**CBG**	Mouse skin melanoma cells	Unknown	In vitro	31.31 µg/mL	↓ Proliferation	N/A	[145]
**WIN 55,212–2 (CB1/CB2 agonist)**	COLO38, SK-MEL-28, OCM-1	MPG antigen (COLO38), HLA-allotyped, BRAF, CDK4, EGFR, PTEN, TERT, TP53 (SK-MEL-28)	In vitro	500 nM, 2 µM, 5 µM.	↓ Cell growth ↑ Apoptosis	CB1 independent affect, CB2 independent affect, VR1 independent affect	[146]
	B16, A375, MelJuso	Spontaneous mouse mutant (B16), BRAF, CDKN2A and TERT (A375), HRAS and NRAS (MelJuso)	In vitro	100 nM	↓ Cell viability ↓ Cell proliferation No effect on mouse melan-c and human Hermes 2b healthy melanocytes	CB receptor-dependent affect for B16 and A375, CB1, CB2, Comparable CB1 expression in mouse melan-c and human Hermes 2b healthy melanocytes	[144]
	B16 injected immune-deficient nude mice and C57BL/6 mice	Spontaneous mouse mutant	In vivo	50 µg/day	↓ Metastasis ↓ Tumour vascularisation ↓ Metastatic nodules in liver and lungs ↑ Apoptosis ↓ Tumour volume Independent of immune response Inhibition of cell cycle at G1-S transition ↓ Tumour proliferation Akt dependent inhibition	CB1, CB2, Comparable CB1 expression in mouse melan-c and human Hermes 2b healthy melanocytes	[144]
**JWH-133 (CB2-selective agonist)**	A2058	BRAF, TERT, TP53, TP63	In vitro	10 µM	↓ Transendothelial migration ↓ Adhesion to brain endothelial cells Involvement of Gi/Goα subunits Downregulation of ICAM, VCAM and MMP	CB2 GPR55 and GPR119 independent	[147]
	B16 injected Immune-deficient nude mice and C57BL/6 mice	Spontaneous mouse mutant	In vivo	50 µg/day	↓ Tumour volume ↓ Tumour vascularisation ↑ Apoptosis ↓ Tumour proliferation ↓ Cell cycle at G1-S transition	CB1, CB2, Comparable CB1 expression in mouse melan-c and human Hermes 2b healthy melanocytes	[143]
	OCM-1A, COLO38	BRAF, CDKN2A and TP53 heterozygous (OCM-1A) MPG antigen (COLO38)	In vitro	500, 2 and 5 µM	No effect	CB1, CB2	[143]
**AM251 (CB1 receptor antagonist)**	HT168-M1	HLA-DR antigen	In vitro	1–10 µM	↑ Apoptosis ↓ Cell cycle at G2/M	CB1	[148]
	A375	BRAF, CDKN2A and TERT	In vitro	6 µM	No effect	CB1	[149]
	OCM-1A, COLO38	BRAF, CDKN2A, TP53 heterozygous (OCM-1A), MPG antigen (COLO38)	In vitro	500, 2 and 5 µM	No effect	CB1	[146]
**AM630 (CB2 receptor antagonist)**	OCM-1A, COLO38	BRAF, CDKN2A, TP53 heterozygous (OCM-1A), MPG antigen (COLO38)	In vitro	1 µM	No effect	CB2	[146]
**Endocannabinoids**							
**PEA**	B16 in C57BL/6 mice	Spontaneous mouse mutant	In vitro	1, 10 and 20 μM	↑ Apoptosis ↓ Cell viability ↑ Cytotoxicity	CB1	[150]
**AEA**	A375	BRAF, CDKN2A and TERT (A375)	In vitro	0.1–100 mM	↓ Cell growth ↓ Cell viability ↑ Cytotoxicity ↑ Caspase-dependent apoptosis Via FAAH inhibition Mitigated by COX-2 and LOX inhibition Possible role of lipid raft and GPR55	CB1, GPR55	[149]
	HT168-M1	HLA-DR antigen	In vitro	1–10 μM	↑ Apoptosis ↓ Cell growth ↓ Metastasis ↓ Migration ↑ Necrosis Cell-cycle Arrest at G2/M	CB1	[148]
	HT168-M1 in SCID mice	HLA-DR antigen	In vivo	10–30 μM	↓ Migration	CB1	[148]
**Met-F-AEA (stable AEA analogue)**	HT-168-M1, WM35, HT199	HLA-DR antigen (HT-168-M1), BRAF (WM35), BRAF, TP53 (WM983B)	In vitro	1–10 µM	↓ Proliferation	CB1	[148]
	HT168-M1 in SCID mice	HLA-DR antigen	In vivo	0.24 or 1.2 mg/kg	↓ Cell growth ↓ Colonization ↓ Liver colonization ↓ Metastasis ↓ Migration No effect on tumour growth	CB1	[148]
**Combined**							
**THC + CBD (Sativex)**	A375, SK-MEL-28, CHL-1	BRAF, CDKN2A and TERT (A375) and BRAF, CDK4, EGFR, PTEN, TERT, TP53 (SK-MEL-28), CDKN2A, MAPK3, TERT, TP53 (CHL-1)	In vitro	1:1 ratio of THC:CBD from 0.5–2.5 μM ideal dosage ratio selected at 1 μM THC+1 μM CBD	↑ Apoptosis ↑ Beclin1 and Ambra1-independent autophagy Independent of BRAF mutational status ↓ Cell viability	Not assessed	[132]
	CHL-1 in athymic nude mice	CDKN2A, MAPK3, TERT, TP53	In vivo	7.5 mg kg^−1^ of 1 μM THC + 1 μM CBD	↑ Apoptosis ↑ Autophagy ↓ Tumour growth	N/A	[141]
**THC + Trametinib**	WM35, A375, SK-MEL-28	CD271 exogenously overexpressed (WM35) BRAF, CDKN2A and TERT (A375) and BRAF, CDK4, EGFR, PTEN, TERT, TP53 (SK-MEL-28)	In vitro	5 µmol L^−1^ THC + 16 nmol L^−1^ Trametinib	↓ Invasion ↓ Metastasis ↓ Viability	N/A	[151]
	A375 in Zebrafish	BRAF, CDKN2A and TERT (A375)	In vivo	5 µmol L^−1^ THC + 16 nmol L^−1^ trametinib	↓Autophagy ↓ Invasion ↓ Metastasis ↓ Viability	N/A	[151]
	OCM-1A, COLO38	BRAF, CDKN2A, TP53 heterozygous (OCM-1A), MPG antigen (COLO38)	In vitro	500 nM, 2 µM, 5 µM	↑ Apoptosis ↓ Cell viability Via lipid raft machinery Involves cleavage of caspases 9 and 7, ERK phosphorylation	CB1 independent affect, CB2 independent affect, VR1 independent affect	[146]
**WIN 55,212–2 + AM251 (CB1 receptor antagonist) + AM630 (CB2 receptor antagonist)**	OCM-1A, COLO38	BRAF, CDKN2A, TP53 heterozygous (OCM-1A), COLO38	In vitro	WIN 500nM + AM251 1 µM + AM630 1 µM WIN 5 µM + AM251 1 µM + AM630 1 µM	↓ Cell viability	CB1, CB2	[146]
**WIN 55,212–2 + AM251 (CB1 receptor antagonist)**	OCM-1A, COLO38	BRAF, CDKN2A, TP53 heterozygous (OCM-1A), COLO38	In vitro	WIN 500nM + AM251 1 µM + AM630 1 µM WIN 5 µM + AM251 1 µM + AM630 1 µM	↓ Cell viability	CB1, CB2	[146]
**WIN 55,212–2 + AM630 (CB2 receptor antagonist)**	OCM-1A, COLO38	BRAF, CDKN2A, TP53 heterozygous (OCM-1A), COLO38	In vitro	WIN 500nM + AM630 1 µM WIN 5 µM + AM630 1 µM	↓ Cell viability	CB1, CB2	[146]
**WIN 55,212–2 + SB36679 (VR1 receptor antagonist)**	OCM-1A, COLO38	BRAF, CDKN2A, TP53 heterozygous (OCM-1A), COLO38	In vitro	500 nM, 5 µM (WIN 55,212-2) + 20 nM (SB36679)	↓ Cell viability	VR1	[146]
**AM251 (CB1 receptor antagonist) + AM630 (CB2 receptor antagonist)**	OCM-1A, COLO38	BRAF, CDKN2A, TP53 heterozygous (OCM-1A), COLO38	In vitro	1 µM of each	No effect	CB1, CB2	[146]
**JWH-133 + ACEA**	HCmel12 and B1	DMBA-induced HGF-CDK4R24C (HCmel12) and spontaneous mouse mutant (B16)	In vitro	500 nM, 2 µM, 5 µM of each	No effect	CB1, CB2	[142]
**AM251 (CB1 receptor antagonist) + Celecoxib**	A375	BRAF, CDKN2A and TERT (A375)	In vitro	0.1–10 µM (AM251) + 0.1–100 µM (Celecoxib)	↑ Apoptosis ↓ Cell growth ↑ Cell-cycle arrest ↓ Expression of antiapoptotic BCL2 and surviving protein expression ↑ Expression of proapoptotic BAX gene ↓ Proliferative at the G2/M transition	CB1, GPR55, TRPA1, and COX-2 in the AM251	[152]
**PEA + URB597 (FAAH inhibitor)**	B16 and MZ2-MEL.43	Spontaneous mouse mutant (B16), NRAS, RAF1 heterozygous (MZ2-MEL.43)	In vitro	10 μM (PEA) + 10 μM (URB597)	↑ Apoptosis ↓ Cell growth ↑ Necrosis ↓Viability Cytotoxicity may involve PEA regulatory affects	Potential weak TRPV1 activation CB1, PPARα, PPARγ and GPR55 independent affect	[150]
	B16 in C57BL/6 mice	Spontaneous mouse mutant	In vivo	Both 10 mg/kg/day	↑ Apoptosis ↑ Necrosis ↓ Tumor volume ↓ Tumor progression Angiogenesis unaffected	Potential weak TRPV1 activation CB1, PPARα, PPARγ and GPR55 independent affect	[150]
**Met-F-AEA (stable AEA analogue) + AM251 (CB1 receptor antagonist)**	HT168-M1	HLA-DR antigen (HT-168-M1)	In vitro	5 µM Met-F-AEA + 1–10 µM AM251	↑ Apoptosis ↓ Cell growth ↓ Cell migration ↓ Metastasis ↑ Necrosis Cell-cycle arrest at the G2/M transition	CB1	[148]
**AEA + URB597 (FAAH inhibitor)**	A375	BRAF, CDKN2A and TERT (A375)	In vitro	0.1–100 mM (AEA) + 1 µM (URB597)	↓ Cell viability	CB1, GPR55, TRPV1 independent	[149]

Cell types are as follows: A375 (human, primary amelanotic melanoma), SK-MEL-28 (human, primary cutaneous), CHL-1 (human, metastatic pleural effusion), HCmel12 (mouse, primary), MelJuso (human, primary cutaneous melanoma), B16 (mouse), COLO38 (invasive adherent metastatic), OCM-1 (uveal non-metastatic), A2058 (human, secondary), B16F1 (mouse), HT168-M1 (mouse, high liver metastasis A2058 variant), OCM-1A (Human, secondary uveal melanoma), WM35 (human, primary cutaneous melanoma), WM983B (human, secondary inguinal lymph node) and HT199 (high liver and lung metastasis).

### 5.6. Whole Cannabis Considerations 

There are many complexities to the landscape of cancer and the endocannabinoid system. The rich network of receptors and enzymes that comprise intricate molecular signaling pathways within the totality of body system, as well as the individual micro-landscape of tumors themselves have yet to be fully elucidated. Previous efforts have revealed significant inconsistencies on multiple fronts, demonstrating variability in the impacts of individual synthetic cannabinoids, dual administration of these cannabinoids and whole cannabis extracts (Table 3 and Table 4). Each of these groups have several points to consider in their potential effective impacts for the treatment of cancer as a wider concept and further toward melanoma alone. 

Current pharmacological practises tend to neglect the synergistic nature of intricate plant products. The application of whole cannabis extracts to a variety of different cancers has an intriguing response; some cannabis chemovars demonstrated more effective anti-cancer capacity toward specific types of cancer than others [165]. Some of the variants demonstrated higher effectiveness against cancer overall, while others were shown to be highly effective for one cancer type. While it was commonly seen that the more effective iterations had higher proportions of THC, this was not the determining factor for their anti-cancer capacity. Conversely, whole extracts tended to outperform the administration of THC alone [140]. Interestingly, this trend in higher combined effectiveness extended to WIN 55,212-2, a highly potent synthetic analogue of THC. Researchers showed that melanoma cell lines have reduced viability when exposed to a combination of CBD and THC and were more effective than those exposed to only THC [166]. 

Medicinal extracts of cannabis and sole cannabinoid administration have shown variable efficacy in the treatment of several cancer types in addition to the aforementioned anti-melanoma actions of THC and CBD. For instance, cervical cancer cell lines have been shown to have significantly higher reduction of cell viability and increased instances of cell death when treated with CBD alone compared to whole cannabis extracts [167]. LNCaP androgen-responsive prostate carcinoma has been shown to have inhibited cell growth, suppressed pro-inflammatory cytokines and induced apoptosis when treated with high CBD low Δ^9^-THC cannabis extracts [95,133]. Furthermore, PC-3 prostate adenocarcinoma and malignant melanoma cell lines A375 have increased cell death when exposed to high Δ^9^-THC cannabis extracts [165].

Thus, synergistic effects of multiple cannabinoids in whole cannabis extracts have more diverse cytotoxic capacity than cannabinol alone treatments [16,140]. Complete cannabis extracts have demonstrated greater anti-tumour effects and fewer side-effects compared to that of cannabinol. This is thought to be the “entourage effect” (EE), where additional cannabinoids such as cannabichromene (CBC) and cannabigeriol (CBG) have a synergistic role in conjunction with THC and CBD for the treatment of disease [167]. Additionally, other minor components, such as terpenoids, flavonoids and other phytocompounds have been shown to have variable alterations on the efficacy of the main cannabinoids through complex interactions in the endocannabinoid system (ECS) not directly pertaining to CB receptor interaction [168]. Impacts of any of these phenolics may vary greatly dependent on the type of cancer cell line that is being treated and many yet unknown cellular, genetic and environmental impacts [169]. 

### 5.7. The Entourage Effect

It has been shown that CBD modulates the more negative side-effects of THC through interactions with the TRPV1 receptor [17,170]. However, impacts of cannabis chemovar potency and response in individual patients is not exclusive to the concentration nor the pure application of the major cannabinoids as indicated in the section above [140,171]. Complex interactions between other minor cannabinols and terpenes have variable impacts on the quality and response to cannabis product treatment [16]. These interactions and the true scope of impact for EE are under continued investigation. Thus far, the pathways by which terpenoids may mediate ECS tone are limited.

However, terpenoid related synergistic interaction is not a result of TRPA1 or TRPA2 modulation [172] nor through direct binding interactions to CR 1 and 2 [166]. Knowledge of the mechanisms by which terpenoids and other polyphenols interact within the ECS are limited. Investigation of the interactions between phytocannabinoids and caryophyllane sesquiterpenes demonstrated that there was an underlying impact related to the presence of terpenes and CBC on the cytotoxic capacity of organic hemp extracts against breast cancer MDA-MB-468 cells [173]. CBD was the primary agent to which the cytotoxicity of these extracts was attributed, via mediation of the CB2 receptor. However, the authors expressed that the role of caryophyllane sesquiterpenes should not be overlooked and minor terpenoids should be further considered [173]. 

The lack of consistent evidence into the role of EE is contributed to by a lack of classification standards of extracts, leading to potentially massive variability in expression of minor biocompounds across past studies. Furthermore, individual response to cannabis extracts is also known to be variable from one person to another when administered with the same chemovar of cannabis [174]. Further mechanisms involved in this action could be related to a deficit in endocannabinoid tone [123]. There may be a homeostatic balancing in individuals administered the diverse chemical profile of cannabis where specific needs and deficits in the endocannabinoid system are regulated by the competitive and synergistic effects of the cannabinoids. A complex interaction of enzymatic and ligand-based interactions systemically modulates an extensive range of regulatory functions within the body, especially those pertaining to pain, inflammation and regulation of emotion [74,175]. It is incredibly challenging to categorise and attribute these effects without sufficient characterisation of cannabis chemovars and markers of individual patient endocannabinoid tone. 

The endocannabinoid system is involved in a deluge of different pathways for the growth, metastasis, angiogenesis and tumorigenesis processes of cancer, the impacts of which may hold key inspiration for melanoma treatment development [176]. Standardising chemical profiles of cannabis and in investigation into markers for individual ECS tone warrant in-depth examination in future research.

### 5.8. Terpenoids 

Although EE was originally assumed to consist of only the influences of other known minor cannabinoids, it has now been suggested that there is a potential role of the major monoterpenoid and sesquiterpenoid compound classes of *C. sativa* as well. Over 100 terpenoid type compounds have been isolated from cannabis extracts [177]. These are hydrocarbons structurally defined by groups of five-carbon structures (isoprene units), whose number defines their structural grouping [177]. Monoterpenes such as β-myrcene, α-pinene, β-pinene, limonene, terpinolene and linalool comprise the most prevalent terpenes identified from cannabis plant samples. Additional prevalent sesquiterpenes include β-caryophyllene and α-humulene [178]. Several of these compounds are known to induce anti-cancerous effects (Table 3) as well as having a range of adjunctive applications. Such applications warrant further exploration for direct applications of these individual components for supportive management and direct support of melanoma patients particularly in the scope of commercial products like PhytoFacts^®^ [179]. Studies pertaining to the direct treatment of melanoma cell lines with terpenes in vitro and in vivo demonstrate inhibition of cellular growth and antimetastatic activity are detailed in Table 3. 

### 5.9. Other Polyphenols of Cannabis 

Due to the limitations of current drug treatments for melanoma, it is imperative that new low toxicity drug options with existing efficacy be employed to improve patient morbidity and mortality. Studies addressed in the below sections demonstrate that naturally based drugs like polyphenols hold existing potential in the sphere of melanoma prevention, treatment and palliative care. They are the most expansively studied plant secondary metabolites and have shown intriguing disease treatment potential in recent years [180,181,182,183,184,185,186]. 

Polyphenols are biocompounds of great medicinal interest for the treatment of heart disease, diabetes, metabolic disorders and cancer due to their well-known anti-inflammatory, antioxidant, anti-bacterial and chemotherapeutic properties [180,181,182,183,184,185,186]. They are characterized structurally by multiple phenolic rings, lack of a nitrogen-containing group and the presence of one or more hydroxyl groups. The formation of these secondary metabolites is performed as a protective mechanism by the plant against UV light damage, insect or fungal attack and a multitude of abiotic stress conditions. 

Several classes of natural polyphenols have well supported medicinal relevance, especially in the treatment and prevention of cancer [182,183]. Cannabinoids, flavonoids and stilbenes are of foremost interest in melanoma treatment [187,188]. 

Other natural polyphenolic compounds have emerging utility as nutraceuticals and have been shown to have anti-cancer action in vivo and in vitro [22,23,180,181,184,185,186,189,190,191,192,193,194,195]. 

Polyphenols that demonstrate high applicability to the treatment of melanoma present in cannabis have been selected for examination in this review. 

Flavonoids, such as catechins found in green tea and stilbenes for example, resveratrol found in red wine, provide an intriguing base point in future drug discovery [188,195,196,197,198,199]. This is due to many disease-preventative and combative impacts of these compounds including their anti-microbial and anti-inflammatory impacts as well as strong applicability to the treatment of melanoma. Of interest is their antioxidant, anti-invasive, anti-tumorigenic and attractive potency against melanoma [188,200,201].

Flavonoids and stilbenes such as apigenin, chrysin and piceatannol among others have strong preventative, anti-inflammatory and specific anti-tumour action [188,189,202,203,204,205,206]. Including apoptotic, cell cycle regulatory, anti-angiogenic, anti-invasive and anti-metastatic abilities demonstrated with in vitro assays. Several of these polyphenols have demonstrated efficacy as drugs against melanoma and are emerging as suggested adjunctive therapies for melanoma treatment; addressed below in Table 4 and Section 6. 

Flavonoids and stilbenes have previously been examined in the treatment of several cancers and other diseases [197]. They have shown various degrees of promise and yet lack efficacy due to poor bioavailability, high dosage requirement and potential toxicities when administered over long periods at higher dosages [204,207,208]. Previous studies have sought to improve the efficacy of polyphenolic compounds by administering them in combination with each other [209,210], by chemically altering their side chains to improve solubility [211] or through other mechanisms such as nanoencapsulation. 

Combinations of flavonoids, such as quercetin, epigallocatechin gallate (EGCG) and genistein, as well as flavonoids with nonflavonoids such as non-cannabis stilbene resveratrol, have shown increased efficacy compared to their individual administration in cell viability assays [209,210,212]. 

Combinations of apigenin and quercetin have been shown to be more effective in the inhibition of lung metastasis of melanoma in a B16-BL6 murine model [188]. Combined use of polyphenols in vivo and in vitro for the treatment of cancer may be a promising venture for drug improvement.

## 6. Application of Cannabis Flavonoids in Melanoma 

### 6.1. Cannflavins

Cannflavin A (CFL-A), B (CFL-B), C (CFL-C) and isocannflavin B (IsoB) are prenylated flavonoids unique to cannabis. Preliminary research has suggested CFL-A and CFL-B as strong anti-inflammatory agents [213]. Such anti-inflammatory action through the inhibition of prostaglandin E2 (PGE2) was demonstrated in the mid 1980’s [214]. 

Additional inflammation modelling studies have examined other facets of this anti-inflammatory action further, demonstrating inhibition of membrane cell-free microsomal prostaglandins [214,215] synthase-1 (mPGEs-1) and 5-Lipoxygenase (mPGEs-5) [216]. These membrane-bound PGE2 synthases are synthesized via the cyclooxygenase 1 (COX-1) and 2 (COX-2) mediated pathways from the endocannabinoid precursor arachidonic acid. Upregulation of COX-2 pathway related genes and a resultant increase in PGE2 are associated with increased mutagenesis and invasion of melanoma cells in vitro. Inhibition of these pathways are thus an attractive target for reduction of melanoma metastasis. 

CFL-A was demonstrated to elicit direct inhibition of COX pathways. Furthermore, mPGEs-1 is associated with the growth, invasiveness and survival of melanoma B16 cells [217]. Suppression of this prostaglandin was associated with significantly higher incidence of apoptosis and reduced cell survival in melanoma cells [218]. There is a lack of evident literature on the action of any cannaflavin for the direct treatment of melanoma clinically or in vivo. However, this avenue of research may provide interesting anti-inflammatory and COX mediated anti-cancer implications in future research. 

### 6.2. Apigenin 

The flavonoid 4′, 5, 7- trihydroxyflavone (apigenin) has intriguing potential for the treatment of cancer due to its multiple treatment complementary effects. It is known to have significant anti-inflammatory and antioxidant capacity, a common feature of the polyphenolic compounds discussed herein.

There have been several in vivo and in vitro studies into the treatment of melanoma that indicate apigenin as a strong candidate for further drug evaluation and treatment prospects, namely, its notable anti-mutagenic, anti-metastatic and anti-invasive properties [189]. These aspects are interesting regarding both its potential preventative administration, such as in high-risk individuals as well as an ongoing sole or combined treatment option for metastatic melanoma. Indeed, in highly invasive B16F10 melanoma cells, apigenin was demonstrated to have significant inhibition of angiogenesis and metastasis in vivo. Additionally, a dose dependent time delay in the progression of these tumours was observed. The anti-invasive capacity of apigenin is at least in part attributed to suppression of constitutive activation of signal transducer and activator of transcription 3 (STAT3). STAT3 is found to be continuously active in melanoma cells and has a metastasis-promoting impact through the regulation of various genes. Suppression of STAT3 has additional impacts on immune regulation via the upregulation of immunosuppressive genes and the downregulation of pro-inflammatory markers [209]. Furthermore, factors such as vascular endothelial growth factor (VEGF) have increased expression, resulting in a stimulatory effect on angiogenesis associated with higher instance of metastasis. Additional oncogenic factors are employed with relation to STAT3 which together promote the growth of melanoma cells, their migration and survival. Targeting these processes through the administration of apigenin has exciting potential for survival improvement of metastatic melanoma.

Further studies that support the efficacy of apigenin for the treatment of cancer show that cell growth is inhibited in numerous cancer cell types. These include breast cancer where there is a promotion of apoptotic pathways via the induction of p53 and p21, as well as STAT3 and nuclear factor kappa light chain enhancer of activated B cells (NFκB) [219,220]. Similarly, in prostate cancer there is a downregulation of genes pertaining to NFκB, namely cyclin DI and COX-2 [221]. VEGF is also hindered as a consequence of apigenin administration in prostate cancer [222]. In melanoma cell lines A2058 and A375, apigenin reduced expression of pro-cancerous pathways through reduction of ERK 1/2 and Fas-Associated Factors (FAF). For uveal melanoma SP6.5 and C918, apigenin acts by suppressing P13/akt and ERK 1/2 pathways [223]. For A375 and C8161 cell lines, a suppression of cell growth mediated by G2/M cell cycle arrest [224] apigenin induced apoptosis via inhibition of the P13K/Akt/mTOR pathway. Additionally, there was a reduction of COX-2 activity in the epidermis of apigenin-treated mice [10,14,225]. Moreover, fewer A375 and C8161 tumors were observed to develop in these mice. 

Apigenin has been explored as a dietary additive and supporting supplement to existing cancer drugs, although limited by its poor solubility resulting in limited absorption and bioavailability. However, it may bioaccumulate in tissues as an ongoing chemoprotective agent broadening its use. 

### 6.3. Chrysin

Chrysin has promise as a chemopreventative and therapeutic agent against cancer. It has been shown to inhibit growth of cancer cells via the induction of apoptosis and cell cycle arrest in the G2/M phase of B16F10 melanoma cells [177]. It has also demonstrated anti-proliferative action against human cutaneous melanoma as well as inhibition of angiogenesis in other tumour types [226]. More exploration of in vivo response for melanoma is needed for chrysin to elucidate its capacity in complex living tumor environments. 

### 6.4. Ferulic Acid 

3-methoxy-p-coumaric acid (ferulic acid) has had growing interest for its chemopreventative, anti-inflammatory and cosmetic properties which have encouraged its addition to a multitude of skin treatment products [204,227,228,229,230,231]. It has been shown to decreases the cell viability of B16F10 murine melanoma cells to 40% in treatments of 1000 µg/mL. Furthermore, ferulic acid may induce melanogenesis, increasing the range of melanin production two to three times the amount compared to control conditions [227]. 

Converse to this melanogenic effect, at a 20 µg/mL concentration, ferulic acid has been shown to decrease intracellular tyrosinase activity resulting in lower melanin production, suggesting a potential whitening effect for skin [228]. Inhibition of TRP-1 and TRP-2 activity as well as related transcription factor microphthalmia transcription factor (MITF) is implicated as the mechanism of tyrosinase reduction in B16F10 melanoma cells treated with ferulic acid. 

Additional mechanistic studies have implicated specific inhibition of casein kinase 2 (CK2) mediated phosphorylation in the melanogenesis pathway [227]. Interestingly, increased concentrations of CK2 in the tumour environment have been implicated in mechanisms of pro-migratory, tumour-promoting and even direct drug resistant characteristics of cancer [227,232]. Thus, compounds that are demonstrated to regulate CK2 may have significant benefit to overcoming the drug-resistant characteristics of melanoma [228,233]. Taken together the research suggests that ferulic acid may regulate tyrosinase activity, inducing pathways of melanogenic or whitening effects dependent on concentration, that has further mechanistic implications for the regulation of melanoma tumorigenesis. 

### 6.5. Genistein

Genistein is known to influence the migration, proliferation and invasive capacities of melanoma. In B16F10 human melanoma cells, genistein has been shown to illicit morphological changes to the cancer that hinder its metastatic ability [234]. Additionally, genistein presents with several other anti-cancer, antioxidant and anti-inflammatory mechanisms that make it an attractive drug target [195,202,234,235]. 

### 6.6. Luteolin

Luteolin is an anti-metastatic agent against melanoma and inhibits the cellular proliferation of A375 and B16F10 melanoma cell lines [236]. This flavonoid significantly impacts the migration, invasion, tube formation and adhesions of these cell lines. Additionally, luteolin is involved in angiogenic targeting of tumoral cells [198]. However, further exploration particularly in living models is necessitated for the true effectiveness of luteolin in melanoma. 

### 6.7. P-Coumaric Acid 

P-coumaric acid (p-CA) has substantial evidence for its anti-melanoma apoptosis and cell cycle dysregulation activities [233,237] in A375 and B16 cell lines. Apoptosis induction via upregulation of Apaf1 and Bax in addition to downregulation of Bcl-2 upon p-CA administration were associated with higher production of cytoplasmic cytochrome c (Cyto-c), cleaved caspase-3 and -9. Cell cycle dysregulation via down regulation of proteins Cyclin-dependent kinase 2 (CDK2) and cyclin A are also features of p-CA administration for the aforementioned cell lines. Additional impacts of p-CA include its antioxidant, anti-inflammatory and competitive inhibition of tyrosinase/melanogenesis action [233,237,238,239,240]. 

### 6.8. Quercetin

Quercetin has been shown to induce apoptosis in melanoma across several studies [241,242]. For example, 50 μg/mL of quercetin was shown to induction apoptosis and anti-proliferative mechanisms in B16 murine melanoma cells resulting in a 75% reduction cell viability [210]. Furthermore, a dose-dependent reduction in cell viability for A375SM melanoma was observed at concentrations above 40 µM. A reduction of up to 32% viability through the induction of apoptosis was observed at the highest assessed concentration of 200 µM for this study. Cell proliferation was also markedly reduced at 27% for 80 µM [211]. However, at a dosage of 20 µM, an increase of 109% cell viability was observed in this study. Additionally, for the other assessed melanoma cell line, A375P, had there was no significant reduction in cell survivability or proliferation [211]. Conclusive evidence explaining this disparity has yet to be elucidated. However, a follow-up in vivo study in mouse models of A375SM demonstrated that quercetin was able to induce apoptosis and lower cancer cell growth through interference with the JNK/P38 MAPK signalling pathway [243]. Differential impacts on the concentration of quercetin administered and subsequent cancer promoting or reducing effects were investigated in an additional study comparing 2D and 3D spheroids. MCM DLN and 1205Lu 3D spheroids demonstrated increased cell proliferation in low concentrations of quercetin (0.4 µM–6.3 µM) [244]. 

Conversely, higher dosages greater than 12.5 µM had significant apoptotic effects for 1205Lu, MCM 1G, and MCM DLN cell types. This difference in impact was attributed to regulation of the Nrf2 pathway, including the activation of ERK and NF-κB kinases and mediation of ROS. Ongoing ERK activation is necessary for the metabolic regulation of these cancer cells. At high doses of quercetin, ERK and NF-κB activation was attenuated resulting in cell death cascades and correlative reduction in cell viability. Furthermore, higher doses of quercetin are thought to induce oxidative stress in cancer cells, contributing to an apoptosis-inducing environment [244]. Quercetin has increasing acclaim for its anti-melanoma impacts in vivo at these higher concentrations. High dose nutritional supplementation of quercetin in cancer suffers is a potential avenue for its administration in the treatment of melanoma.

### 6.9. Rutin 

Rutin is shown to have anti-melanoma features in previous studies of its application [186,245,246,247]. For example, induction of pro-apoptotic mechanisms and reduction in proliferation were observed in B164A5 murine cells at 100 µM doses of rutin [247]. Furthermore, in vivo reduction of 71% of nodular tumour presentation in the lungs of B16F10 mice (dosed orally with rutin) was recorded as early as 1995 [246]. Most interestingly, rutin has also been employed in combination with BRAF inhibitor Vemurafenib; targeting Vemurafenib resistance in SKMEL-28 resistance induced cells. Beyerstedt and colleges demonstrated that 100 µM of rutin co-administered with 3 µM of Vemurafenib resulted in 60% cell death of SKMEL-28 [248]. Additionally, rutin is a protein disulfide isomerase (PI) inhibitor, an enzyme highly expressed in malignant melanoma [248]. PI inhibition may account for the aforenoted reductions in proliferation and the circumvention of Vemurafenib resistance. Thus, PI could be an intriguing receptor target for rutin mitigated BRAF inhibitor resistance for future studies. Additionally, coadministration studies which apply rutin and other combined therapies such as those listen in Table 1 would greatly expand data relating to the efficacy of its adjunctive application against melanoma.

## 7. Conclusions

In conclusion, there is a complex array of effects that polyphenolic and cannabinoid compounds elicit in relation to melanoma. Multiple biochemical and genetic cascades are regulated through the presence of these natural substances [241,242]. Polyphenolic compounds emergingly demonstrate a significant capacity to mediate many of the impacts of cancer, including pain, inflammation and invasiveness. Combined administration of polyphenol compounds has shown existing promise for improvement of potency and bioactivity of these substances [249,250]. To combat the complexity of cancer, new pharmacological perspectives are necessary. Accordingly, plant polyphenols, particularly those of cannabis provide a deep well of structural potential for the emergence of novel drugs with multi-applicability to the total sphere of cancer treatment. This is merely the budding tip of biocompounds available for exploration in plant-based medicine and is a substantive base for future research. 

## Figures and Tables

**Table 3:** Effects associated with the administration of Terpenoids in the treatment of melanoma cells in vitro and in vivo.

Terpenoid	Cell Type	Effect	In Vitro/ In Vivo	Reference
**α-Pinene**	B16F10 murine melanoma cell line syngeneic in C57Bl/6 mice	↓ Lung tumor nodules ↓ Metastatic activity ↑ ROS production ↑ Early apoptotic features such as: DNA fragmentation Phosphatidylserine on the cell surface Disruption of mitochondrial membrane potential	In vitro/In vivo	[153]
**Luteolin**	SK-Mel2, A375 and SK-Mel28, WM3211 Athymic nude mice (Strain 490)	↓ Cell growth via: Extracellular matrix, oncogenic signaling, and immune response pathways Not through ROS induction	In vitro/In vivo	[154]

**Table 4:** Phenol treatments on melanoma cell lines in vitro and in vivo. Oxygen consumption rate (OCR), extracellular acidification rate (ECAR).

Phenol	Cell Lines	Anticancer Effect	Mechanism of Action	In Vivo/In Vitro	References
**Apigenin**	A375SM human melanoma cells	↓ Cell viability ↑ Apoptosis ↓ Tumour growth	↑ Apoptosis via regulating the Akt and mitogen-activated protein kinase signalling pathway	In vitro/In vivo	[155]
**Ferulic Acid**	Melanoma A375, CHL-1, SK-MEL-2, B16F10 cells B16F10 cells in female C57BL mice	↓ Proliferation ↓ Angiogenesis In vivo	FGFR1-mediated PI3K-Akt signalling pathway Blocking of the PI3K-Akt pathway	In vitro/In vivo	[156]
**Quercetin**	B16 and A375 murine model	↓ Tumour growth ↓ Proliferation ↑ Apoptosis	↑ IFN-α and IFN-β expression through activation of RIG-I promoter in B16 cells	In vivo	[157]
	B164A5 murine melanoma cell line	↓ Mitochondrial respiration ↑ Apoptotic ↓ Proliferative	↓ OCR ↓ ECAR Modulated glycolytic and mitochondrial pathways for ATP production	In vitro	[158]
	DB-1	Targetted Tyr+ expressing melanoma cells ↑ Apoptosis	↓GSH ↓ Bio reduction capacity ↑ ROS	In vitro	[159]
	Murine B16-F10 melanoma cells	↑ Melanin production ↓ Cell viability	↑ Activity and synthesis of tyrosinase	In vitro	[160]
	Human melanoma A375 and A2058 cells, and B16F10 cells in male C57BL model	↓ Proliferation ↑ Apoptosis ↓ Migration and Invasive ↓ Metastasis	↓ STAT3 signalling Interfered with STAT3 phosphorylation ↓ STAT3 nuclear localization ↓ A375 tumour growth ↓ STAT3 signalling	In vitro/In vivo	[161]
	A375SM and A375P human melanoma cells	↓ Viability and proliferation of A375SM cells No effect on A375P cells ↓ A375SM tumour volume ↑ Apoptosis	↑ Expression of Bax, phospho-JNK, phospho-p38 and phospho-ERK1/2 Cleaved poly-ADP ribose polymerases ↓ Bcl-2	In vitro/In vivo	[162]
	B16	↓ Proliferation ↓ Proportion of cells in S and G2/M stages of the cell cycle ↑ SubG1 population of treated cells	Cell cycle disruption	In vitro	[163]
		↓ HGF ↓ Cell migration ↓ Metastasis	↓ HGF-induced melanoma cell migration ↓ c-Met homo-dimerization and phosphorylation ↓ c-Met protein expression ↓ Activation of c-Met and downstream Gabl, FAK and PAK Suppression of the HGF/c-Met signaling pathway		[164]

## Data Availability

Not applicable.

## Abbreviations

AJCC	American Joint Committee on Cancer
AEA-N	Arachidonoylethanolamine
2-AG	2-Arachidonoglycerol
CB1	Cannabinoid receptor 1
CB2	Cannabinoid receptor 2
CFL-A	Cannflavin A
CFL-B	Cannflavin B
CFL-C	Cannflavin C
CBD	Cannabidiol
CBG	Cannabigerol
CBN	Cannabinol
CBC	Cannabichromene
CBV	Cannabidivarin
CBL	Cannabicyclol
CBE	Cannabielsoin
CBDA	Cannabidiolic acid
CDKN2A	Cyclin-dependent kinase inhibitor 2A
CDK4	Cyclin-dependent kinase 4
CDK6	Cyclin-dependent kinase 6
CK2	Casein kinase 2
CMM	Cutaneous malignant melanoma
Cyto c	Cytochrome c
CTLA-4	Cytotoxic T lymphocyte-associated protein 4
ECS	Endocannabinoid system
ER	Endoplasmic reticulum
EE	The entourage effect
EGCG	Epigallocatechin gallate
FAAH	Fatty acid amide hydrolase
FABPs	Fatty acid-binding proteins
FAF	Fas-associated Factors
HT	Hypertrophic
HO_2_^•^	Hydroperoxyl
ITM	In-transit melanoma metastasis
IsoB	Isocannflavin B
LDL	Lactate dehydrogenase level
MEK	Mitogen-activated protein kinase
MiRNAs	MicroRNAs
MDM2	Mouse double minute 2 homolog
MITF	Microphthalmia transcription factor
NF1	Neurofibromatosis type 1
NEAs	N-acyleethanolamines
OEA	Oleoylethanolamide
OS	Overall survival
OH	Hydroxyl free radicals
O_2_¯	Anion superoxides
p-CA	p-Coumaric acid
PDI	Protein disulfide isomerase
PD-1	Programmed cell death protein 1
PEA	Palmitoyethanolamide
PGE2	Prostaglandin E2
ROS	Reactive oxygen species
RO_2_^•^	Peroxyl
SCC	Squamous cell carcinoma
STAT3	Transcription 3
THC	Δ9-tetrahydrocannabinol
TRPV1	TRP vanilloid type 1
TRPV2	TRP vanilloid type 2
TRPV3	TRP vanilloid type 3
TRPV4	TRP vanilloid type 4
TRPVA1	TRP ankyrin 1
TRPM8	TRP melastatin 8
UV	Ultraviolet
UVR	Ultraviolet radiation
VEGF	Vascular endothelial growth factor
